# Delivering an Online Cognitive Behavioral Therapy Program to Address Mental Health Challenges Faced by Correctional Workers and Other Public Safety Personnel: Protocol for a Mixed Methods Study

**DOI:** 10.2196/30845

**Published:** 2021-07-22

**Authors:** Nazanin Alavi, Callum Stephenson, Mohsen Omrani, Cory Gerritsen, Michael S Martin, Alex Knyahnytskyi, Yiran Zhu, Anchan Kumar, Jasleen Jagayat, Amirhossein Shirazi, Elnaz Moghimi, Charmy Patel, Yuliya Knyahnytska, Alexander I F Simpson, Juveria Zaheer, Judith Andersen, Alpna Munshi, Dianne Groll

**Affiliations:** 1 Department of Psychiatry Faculty of Health Sciences Queen's University Kingston, ON Canada; 2 Centre for Neuroscience Studies Faculty of Health Sciences Queen's University Kingston, ON Canada; 3 OPTT Inc Toronto, ON Canada; 4 Forensic Early Intervention Service Centre for Addiction and Mental Health Toronto, ON Canada; 5 Department of Psychiatry University of Toronto Toronto, ON Canada; 6 Department of Psychological Clinical Science University of Toronto Toronto, ON Canada; 7 Health Services Sector Correctional Service Canada Ottawa, ON Canada; 8 School of Epidemiology and Public Health University of Ottawa Ottawa, ON Canada; 9 Faculty of Health Sciences Queen's Unviersity Kingston, ON Canada; 10 General Psychiatry Division Centre for Addiction and Mental Health Toronto, ON Canada; 11 Department of Forensic Psychiatry Centre for Addiction and Mental Health Toronto, ON Canada; 12 Institute for Mental Health Policy Research Centre for Addiction and Mental Health Toronto, ON Canada; 13 Department of Psychology University of Toronto Mississauga Mississauga, ON Canada; 14 Canadian Institute for Military and Veteran Health Research Kingston, ON Canada

**Keywords:** mental health, correctional workers, public safety personnel, depression, anxiety, psychotherapy, cognitive behavioral therapy, online, internet, treatment

## Abstract

**Background:**

Public safety personnel have regular and often intense exposure to potentially traumatic events at work, especially workplace violence in the case of correctional workers. Subsequently, correctional workers are at higher risk of developing mental health problems such as posttraumatic stress disorder. Public safety personnel are up to 4 times more likely to experience suicidal ideation, suicidal attempts, and death by suicide compared to the general population. Despite this high prevalence, help-seeking behaviors from public safety personnel are low due to stigma and irregular work hours limiting access to care. Innovative treatments are needed to address these challenges.

**Objective:**

This study will investigate the efficacy of an electronically delivered cognitive behavioral therapy (e-CBT) program tailored to correctional workers’ mental health problems.

**Methods:**

This study is composed of 4 phases. In phase 1, we will interview correctional workers individually and in focus groups to identify personal, social, and cultural factors affecting their mental health and barriers to care. Phase 2 will use the information gathered from the interviews to develop gender- and diagnosis-specific e-CBT modules. These will be presented to a new group of participants who will provide further feedback on their usability and accessibility. In phase 3, we will randomly assign participants to either an e-CBT or treatment as usual arm. The program will be evaluated with validated symptomatology questionnaires and interviews. Phase 4 will use this additional information to fine-tune the e-CBT modules for a larger-scale randomized controlled trial design comparing the e-CBT program to in-person CBT. All e-CBT modules will be delivered through a secure online platform.

**Results:**

The study received ethics approval in December 2020, and participant recruitment began in March 2021. Participant recruitment has been conducted through targeted advertisements and physician referrals. To date, there have been 15 participants recruited for Phase 1, and it is expected to conclude in July 2021, with phase 2 beginning in September 2021. Complete data collection and analysis from all phases are expected to conclude by July 2023. Linear and binomial regression (for continuous and categorical outcomes, respectively) will be conducted along with interpretive qualitative methods.

**Conclusions:**

If proven efficacious and feasible, this e-CBT program can provide a high-quality and clinically validated resource to address the mental health problems of correctional workers. Additionally, findings can contribute to the development of innovative treatments for other public safety professions.

**Trial Registration:**

ClinicalTrials.gov NCT04666974; https://www.clinicaltrials.gov/ct2/show/NCT04666974

**International Registered Report Identifier (IRRID):**

DERR1-10.2196/30845

## Introduction

### Background

Public safety personnel (PSP) have regular and often intense exposure to traumatic events and are at higher risk for developing mental health disorders including posttraumatic stress disorder (PTSD), major depressive disorder (MDD), generalized anxiety disorder (GAD), and drug and alcohol use disorders [1]. PSPs are up to 4 times more likely to experience suicidal ideation, suicidal attempts, and death by suicide compared to the general population [2]. Correctional workers (CWs) are at a particularly elevated risk of developing mental health disorders, as they work in a hostile environment with an increased prevalence of violence [3]. A survey found that over 98% of CWs have had at least one encounter with job-related traumatic events, frequently involving exposure to serious injury or death; casualties; unusual sights, sounds, and circumstances; first-hand knowledge of the victim(s); and threats to their safety and security [4]. It was also found that CWs, on average, will experience approximately 28 traumatic events during their career [4]. Many CWs feel there are not enough organizational policies to debrief following the occurrence of such difficult situations, along with a lack of after-hour counselling, leaving them concerned with the limited support available through their employee assistance programs [5]. The absence of sufficient organizational policies exacerbates the effects of violent incidents on CWs, leading to PTSD being 3 times more prevalent in CWs compared to the general population [6-8]. Therefore, the need to address this mental health crisis in CWs must be a top priority.

Psychotherapy, and more specifically, cognitive behavioral therapy (CBT), is the first-line treatment for various anxiety disorders, including PTSD [9-17]. Psychotherapy can be more cost-effective, decrease relapse prevalence, outperform many pharmacotherapies, and be the intervention of preference for many patients when treating anxiety disorders [18-23]. CBT is one of the most widely investigated and practiced forms of psychotherapy and is effective in reducing the deleterious symptoms of mood and anxiety disorders [24].

While CBT has a well-established efficacy in improving various mental health disorder symptoms, many patients do not receive treatment [25]. This is due to the multitude of barriers to receiving treatment, which include psychological, social, geographical, financial, and systemic factors [26]. Despite the high rate of mental health disorders in PSPs, their willingness to seek treatment is low because of the stigma attached to mental health in their profession [27,28]. Many PSPs feel discredited by seeking treatment for their mental health and feel an expectation from their job to tolerate their symptoms [28,29]. Among those who do seek help, they face unique barriers including irregular work hours, limiting their access to resources otherwise available to the public. In many cases, the publicly available options, such as group CBT, add to the stigma of receiving care, especially in smaller cities.

A promising solution to address these barriers is the asynchronous, text-based, module-driven, diagnosis-specific, electronic delivery of CBT (e-CBT). In recent years, there has been an increased interest in researching the efficacy of e-CBT, due to its ability to address many barriers associated with in-person CBT [30-32]. Delivering e-CBT asynchronously allows for therapy to be accessed at a convenient time for the patient from the comfort of their own home. e-CBT content can be adapted to a multitude of languages and cultures, meeting the needs of more patients. Moreover, the private nature of e-CBT can reduce the stigma of receiving care [28,33]. e-CBT has been shown to effectively treat a variety of mental health disorders, supported by several meta-analyses [34-37]. Regarding depression and anxiety disorders, e-CBT can be efficacious as a singular or adjunctive treatment [38-40]. Additionally, e-CBT has also been shown to effectively address PTSD symptoms [41-45]. These findings are promising as e-CBT techniques are cost-effective, are geographically and temporally accessible, have shorter waiting times for treatment, reduce stigma, increase help-seeking among patients, and can increase treatment adherence [46,47]. More importantly, the treatment outcomes of e-CBT are suggested to be comparable to in-person psychotherapy for individuals with GAD [38]. Regarding scalability, e-CBT can offer up to 70%-80% time savings for therapists [47].

There are many forms of e-CBT, including unguided self-help, guided self-help (standardized program with support from a mental health professional), and individualized therapy (tailors therapy modules to patient needs based on an entry interview) [48-50]. The length and mode of delivery vary between programs; however, they all utilize the same strategies as in-person CBT. While all programs have some form of efficacy, their impact can vary greatly. It has been found that the major factor affecting program efficacy is the level of care provider engagement [51]. While many studies suggest even self-guided content can be beneficial, more therapist engagement is related to higher treatment efficacy [51,52]. Therapy outcomes (ie, symptom reduction, remission) are up to 3 times higher in supervised e-CBT compared to self-guided methods [53]. Therefore, a supervised e-CBT approach is needed to effectively address the mental health needs of this PSP population.

### Objectives

Each phase has a respective objective. In phase 1, the aim is to understand CWs’ mental health challenges and barriers to receiving care. In phase 2, we will create e-CBT modules to address the CW-specific mental health problems found in phase 1. In phase 3, the aim is to evaluate the e-CBT program efficacy compared to treatment as usual (TAU). In phase 4, we will evaluate the e-CBT program efficacy compared to in-person CBT.

## Methods

### Design

This study is split into 4 phases with the first 2 using a qualitative approach to uncover mental health challenges and barriers to care faced by CWs, along with the usability of the e-CBT modules developed. These phases will use qualitative interviews and focus groups to collect data. Phase 3 will use a randomized controlled trial (RCT) design, with participants being allocated to either an e-CBT or TAU arm. In phase 4, an RCT design will be employed again, comparing the efficacy of the e-CBT arm to an in-person CBT arm. This study has been registered with ClinicalTrials.gov (NCT04666974).

### Recruitment

In phase 1, a group of experienced CWs (n=5) will be initially recruited. These CWs will have in-depth knowledge of CW problems and will be chosen in consultation with CW union members. Moreover, members of this research team have extensive experience working with Correction Services Canada and will use their expertise to select suitable participants. Following this, a larger group of CWs (n=40; 20 men, 20 women) will be recruited for individual and focus group interviews. Recruitment will be conducted using flyers (distributed in the workplace), advertisements in specialized journals, union representatives, and word of mouth.

In phase 2, CWs (n=20; 10 men, 10 women) will be recruited using the same methods as in phase 1.

In phase 3, CWs (n=100; 50 men, 50 women) will be recruited using the same materials as in the previous phases; however, participants will also be accepted based on physician referrals.

In phase 4, CWs (n=100; 50 men, 50 women) will be recruited using the same methods as in phase 3. However, an additional step will be taken to ensure eligibility by conducting a comprehensive interview by a psychiatrist, further validated by a structured interview performed by a trained research assistant.

### Inclusion/Exclusion Criteria

Participants will undergo a comprehensive initial mental health assessment by a psychiatrist on the research team. These assessments will occur either in person or through video calls. Diagnoses will be identified using the structured Mini-International Neuropsychiatric Interview (MINI) performed by a trained research assistant and a psychiatric assessment conducted by a psychiatrist on the research team. Inclusion criteria include 18-55 years of age at the start of the study; diagnosis of MDD, GAD, or PTSD according to the Diagnostic and Statistical Manual of Mental Disorders, 5th Edition (DSM-5); competence to consent and participate; ability to speak and read English; and consistent and reliable access to the internet [54]. Exclusion criteria include diagnosis of hypomanic/manic episodes, psychosis, severe alcohol or substance use disorder, and active suicidal/homicidal ideation.

### Phase 1

An expert end-user group of CWs (n=5) will help the research team across all phases of this project. These experts will have in-depth knowledge of CW problems and will be chosen in consultation with CW union members. A new set of CWs (n=40; 20 men, 20 women) will be recruited to participate in individual interviews and then in virtual focus groups through Microsoft Teams as this platform is approved for security reasons by the Queen’s University Health Science and Affiliated Teaching Hospitals Research Ethics Board (HSREB). These interviews will be used to identify the main mental health challenges and the best methods to address them in the context of different groups of CWs (eg, administrative staff, operational workers, direct vs indirect contact with prisoners, working in a prison vs working in probation offices) and investigate the barriers specific to each group. Each individual will be independently interviewed by a trained research assistant hired for the project (60-90–minute duration). Interviews will occur remotely via a secure video conference with Microsoft Teams. Questions will include topics of the level of interaction with prisoners, types and frequency of violence at work, sexual assault, work condition (staffing, shift length, relationship with superiors and co-workers, workload, over-crowdedness), socioeconomic factors (race, gender, language, sexuality, religious discrimination), mental challenges, resources (training, peer support, and professional help) and frequency they use them, reasons for refraining to use resources, preferred method of support (onsite vs offsite, individual vs group), and attitude towards online care (willing to do so and what content they want to be covered). Information gathered through individual interviews will form the basis for further focus group discussions via Microsoft Teams.

Following individual interviews, 5 half-day focus groups (8 participants per group, 2 exclusively female, 2 exclusively male, 1 sex balanced) will be conducted to gather more information regarding issues uncovered in individual interviews. These focus groups will occur remotely via video conference with Microsoft Teams. A list of challenges to be addressed in phases 2-4 will be formed from these interviews. The principal investigator, 1 psychiatrist, 1 psychologist, 1 research assistant, and at least 2 members of the end-user expert group will attend each group meeting to form a better understanding of the problems faced by each group. All interviews and focus group discussions will be recorded and transcribed verbatim for further discussion at the investigative team meetings.

### Phase 2

Following the first round of content development, e-CBT modules will be presented to the end-user expert group and a smaller number of interview/focus group participants (n=20; 10 men, 10 women) for feedback. Each participant will be asked to provide feedback on 1 of the 6 therapeutic modules regarding content, form, presentation, examples, and helpfulness, based on the challenges it tries to address. All feedback will be summarized and compiled by research assistants and discussed in 6 separate focus group discussions attended by the principal investigator, 1 psychiatrist, 1 psychologist, 1 research assistant, and at least 2 members of the end-user expert group. Each participant is requested to attend 2 virtual focus group discussions on the module they have not seen yet or commented on. This is done to ensure each participant can provide feedback on all gender-specific therapy contents. A list of recommended changes to each module will be developed through these discussions. Recommendations will be reviewed by the principal investigator, co-applicants, and the knowledge users for clinical validation. The final changes, compiled by clinicians, will be applied to the e-CBT module.

### Phase 3

CWs (n=100; 50 men, 50 women) meeting the inclusion/exclusion criteria will complete a set of socioeconomic, demographic, and the following clinical questionnaires (primary diagnosis dependent) at baseline: Depression and Anxiety Stress Scale - 42 Item (DASS-42), Quality of Life Satisfaction and Enjoyment Questionnaire (Q-LES-Q), Quick Inventory of Depressive Symptoms Self Report - 16 Item (QIDS-SR16), Patient Health Questionnaire - 9 Item (PHQ-9), Generalized Anxiety Disorder - 7 Item (GAD-7), and PTSD Checklist for the DSM-5 (PCL-5) [55-60]. Questionnaires and e-CBT modules will be completed through the Online Psychotherapy Tool (OPTT; OPTT Inc.), a secure, cloud-based, mental health care delivery, online platform. OPTT can be accessed from various devices (ie, computer, cellphone, tablet) and internet browsers. Following the initial assessment, participants will be randomly allocated to the e-CBT + TAU (n=50; 25 men, 25 women) or TAU (n=50; 25 men, 25 women) arm.

Participants in the e-CBT group will be assigned to 1 of the 6 therapy modules based on their gender and diagnosis (ie, female/male; GAD/MDD/PTSD). All e-CBT sessions will be completed through OPTT. Sessions will be designed to mirror in-person CBT content and consist of approximately 30 slides and interactive therapist videos (40-60–minute completion time), with 12 sessions in total (1 module per week). The modules will focus on standard aspects of CBT (the connection between thoughts, behaviors, emotions, physical reactions, and environment) and incorporate different skills into each session (eg, mindfulness, goal setting, activity scheduling). Each participant will be assigned a specific therapist who will be their care liaison through the program. Each week, the clinician will send the new e-CBT session to the participant through OPTT on a predetermined day of the week. Each of these sessions will include a homework assignment that the participant must send back to their clinician by a predetermined date to gain access to the next session. Upon receiving the homework submission, the clinician will review the participant’s work and provide individualized feedback on their performance. This feedback will be sent back to the participant with their new e-CBT module the following week. Clinician feedback will follow a structured format and take approximately 15 minutes per patient, allowing for increased scalability.

Participants in both arms will complete the symptomology questionnaires every 2 weeks along with 6-month and 12-month follow-ups. Additionally, participants in the e-CBT arm will complete a qualitative questionnaire regarding their experience using OPTT. These questions will relate to the aesthetic appeal of OPTT, the intuitiveness of OPTT, technical support experience, OPTT navigation and simplicity, and the kinds of devices used to access therapy. Information from this qualitative questionnaire will be used to fine-tune the e-CBT modules for phase 4.

### Phase 4

At baseline, participants (n=100; 50 men, 50 women) will complete the same socioeconomic, demographic, and clinical questionnaires as in phase 3 through OPTT. Following the initial assessment, participants will be randomly allocated to either the e-CBT + TAU (n=50; 25 men, 25 women) or in-person CBT + TAU (n=50; 25 men, 25 women) arm. Participants in the TAU arm will continue with current lifestyle choices (ie, physical activity, diet, socialization) and current treatments (ie, pharmacotherapy, meditation) for the duration of the program.

Participants in the e-CBT arm will receive a specific program based on their primary diagnosis and gender (male/female; GAD/MDD/PTSD). Participants will be assigned a specific therapist who will be their care liaison throughout the program. Participants in the in-person CBT arm will receive similar content, homework, and feedback compared to the e-CBT arm. All in-person CBT sessions (60-75–minute completion times) will be delivered by a trained professional.

Participants in both arms will complete the symptomology questionnaires biweekly (same as in phase 3) along with 6-month and 12-month follow-ups. Following week 12 (posttreatment), participants will also complete an additional MINI. Additionally, participants in the e-CBT arm will complete the same qualitative questionnaire relating to their experience using OPTT. These responses will be discussed with the end-user expert group to form recommendations for improving the e-CBT modules through OPTT.

### Training

Therapists will be trained in psychotherapy delivery and supervised by a psychiatrist on the research team who has extensive experience in e-CBT. To ensure consistency of care, therapists will learn a standardized pathway of care and undergo sample training sessions before interacting with patients. Feedback templates will vary between e-CBT modules with therapists providing personalization directly related to their participants’ work. Before submission, all feedback will be reviewed and approved by a psychiatrist on the research team.

### Outcomes

Phases 1 and 2 will be completed using qualitative individual interviews along with focus groups. Phases 3 and 4 will be completed using the quantitative methodology and will be based on the findings from phases 1 and 2.

Phase 1 aims to evaluate mental health challenges and barriers to receiving care faced by CWs. This information will be used to create the e-CBT modules that will be presented to participants in phase 2, where the usability of these modules will be investigated.

In phases 3 and 4, the primary outcome will be comparing changes in symptom severity from baseline to posttreatment between e-CBT and TAU/in-person CBT arms, respectively. This will be evaluated using the DASS-42, Q-LES-Q, QIDS-SR16, PHQ-9, GAD-7, and PCL-5. These questionnaires will be completed biweekly starting at baseline until posttreatment (week 12), followed by 6-month and 12-month follow-ups.

The secondary outcomes of phases 3 and 4 will be evaluating how personal, social, and demographic factors can impact treatment experience. This will be investigated using the data from the qualitative questionnaires.

### Analysis

Qualitative analysis of interview and focus group data (phases 1 and 2) will be performed using an iterative analysis process where investigators will review interview transcripts and search for key phrases, ideas, and themes directly pertaining to the research questions. Similarly, the qualitative comments and feedback obtained from questionnaires in phases 3 and 4 will also be transcribed and analyzed for themes. The information collected in phases 1 and 2 will be used to develop the e-CBT modules, with the information collected through phases 3 and 4 being used to finalize the format of the e-CBT (if necessary).

In phases 3 and 4, the relationship between socioeconomic status, demographic factors, and mental health challenges faced by CWs will be investigated. Questionnaire data will initially be examined for missing, nonsensical, and outlying variables and corrected where possible. Missing data will be treated as missing and not imputed (ie, will be analyzed on a per-protocol basis).

Following this, the symptomatology questionnaires (DASS-42, Q-LES-Q, QIDS-SR16, PHQ-9, GAD-7, and PCL-5) will be analyzed based on score change from baseline to posttreatment, 6-month follow-up, and 12-month follow-up. Questionnaire scores will be calculated for each time point, and then the changes between the times will be assessed using multivariate repeated measures analysis of variance (MANOVA). Comparisons will then be made between the e-CBT and control arms using linear regression models while controlling for demographic variables (eg, age, gender).

Factoring in anticipated dropouts, recruitment numbers have been purposely oversampled. From previous research in similar patient populations, dropouts can be expected to be up to 25% [61]. Given that there are 6 specialized versions of the e-CBT modules with different symptom questionnaires, it is difficult to calculate a single sample size or provide specific power calculations. However, using the PCL-5 as an example as it will be common to all participants, a 10-point change is considered clinically significant [62]. Therefore, a sample size of 30 participants in each arm will be sufficient for detecting significant results with *P*=.05 and a power of 0.8.

### Ethics and Data Privacy

Only the care providers directly involved in participant interaction will be able to access their information. Participant identity will be kept anonymous through randomized participant ID numbers on OPTT. OPTT is compliant with the Health Insurance Portability and Accountability Act, the Personal Information Protection and Electronic Documents Act, and the Service Organization Control - 2. Additionally, all servers and databases are hosted in Amazon Web Service Canada cloud infrastructure, which is managed by Medstack to assure all provincial and federal privacy and security regulations are met. OPTT will not collect any identifiable personal information or internet protocol addresses for privacy purposes. OPTT will only collect anonymized metadata to improve its service quality and provide advanced analytics to the clinician team. All encrypted backups will be kept on secure Queen’s University servers.

## Results

The study received notice of funding acceptance in March 2020. Ethics approval from the Queen’s University HSREB (File Number: 6029966) was obtained in December 2020. Participant recruitment began in March 2021 using targeted advertisement and physician referrals (see the Recruitment section). To date, there have been 15 participants recruited for Phase 1, and it is expected to conclude in June 2021 with phase 2 beginning in September 2021. Complete data collection and analysis from all phases are expected to conclude by July 2023. Focus group analysis described earlier will be conducted after phases 1 and 2, and regression analysis (continuous and categorical outcomes) will be conducted after phases 3 and 4. All procedures and outcomes have been and will be reported using the GUIDED (Multimedia Appendix 1) and TIDieR Report Guidelines (Multimedia Appendix 2).

## Discussion

CWs are at a significantly elevated risk of developing a mental health disorder. Due to stigma, geographic isolation, cost, and irregular work hours, CWs are less likely to seek treatment for their mental health struggles. To address this, innovative treatments are needed. CBT is an effective treatment; however, it is not readily available to CWs due to long wait times. The delivery of CBT through the internet is a promising solution. The outcomes of this study can both improve our understanding of CW mental health challenges and provide an innovative approach to address them. Focus group discussions will explore specific challenges each group of CWs face so therapy modules can be designed and targeted accordingly. The comprehensive mental health evaluations will also provide an invaluable survey of the mental health status of CWs.

If proven effective, e-CBT could be an accessible option for CWs to receive care whenever, wherever, and in any language they need without worrying about the stigma of receiving care. If shown to be comparable to in-person CBT, this method of care delivery could massively expand the therapy capacity in the public sector without sacrificing the quality of care.

This project has the potential to influence clinical care and health care policy by addressing barriers currently preventing CWs from receiving the care they need. This approach would offer monetary savings to the health care system and provide a more equitable and accessible method of delivery. Asynchronous e-CBT takes less time for the clinician to deliver, while still providing comprehensive benefits of therapy with added accessibility and efficiency. This increased efficiency can allow clinicians to be more productive within their clinical time and workload.

## Appendix

Multimedia Appendix 1 GUIDED Report Checklist.

Multimedia Appendix 2 TIDieR Report Checklist.

Multimedia Appendix 3 Funding acceptance letter and reviewer comments from the Canadian Institutes of Health Research.

## Abbreviations

CBT: cognitive behavioral therapy
CW: correctional worker
DASS-42: Depression and Anxiety Stress Scale - 42 Item
DSM-5: Diagnostic and Statistical Manual of Mental Disorders, 5th Edition
e-CBT: electronically delivered cognitive behavioral therapy
GAD-7: Generalized Anxiety Disorder - 7 Item
HSREB: Health Science and Affiliated Teaching Hospital Research Ethics Board
MANOVA: multivariate repeated measures analysis of variance
MDD: major depressive disorder
MINI: Mini-International Neuropsychiatric Interview
OPTT: Online Psychotherapy Tool
PCL-5: PTSD Checklist for the DSM-5
PHQ-9: Patient Health Questionnaire - 9 Item
PSP: public safety personnel
PTSD: posttraumatic stress disorder
QIDS-SR16: Quick Inventory of Depressive Symptoms Self Report - 16 Item
Q-LES-Q: Quality of Life Satisfaction and Enjoyment Questionnaire
RCT: randomized controlled trial
TAU: treatment as usual
