# Supplementary material for: Delivering an Online Cognitive Behavioral Therapy Program to Address Mental Health Challenges Faced by Correctional Workers and Other Public Safety Personnel: Protocol for a Mixed Methods Study
Source: JMIR Res Protoc. 2021 Jul 22;10(7):e30845. doi: 10.2196/30845 (PMC8367142; doi:10.2196/30845)
Supplement: Multimedia Appendix 3 [file resprot_v10i7e30845_app3.pdf]

March/Mars 24, 2020

DC0190GP

Canadian Institutes of Health Research / Instituts de recherche en santé du Canada

Notice of Decision / Avis de décision

Application Number/Numéro de la demande: 433679

Committee Code/Code du comité: MWP

Applicants/Candidats: Dr. Nazanin Alavi; Dr. Alexander Ian Frederic Simpson

With/Avec: Dr. J. Andersen; Dr. C. Gerritsen; Dr. D. Groll; Dr. Y. Knyahnytska; Prof. M. Martin; Dr. A. Munshi; Dr. J. Zaheer

Institution paid/ Établissement payé: Queen's University (Kingston, Ontario)

Title/Titre: Delivering online cognitive behavioural therapy (iCBT) to address mental health challenges in correctional officers and other public safety personnel

Primary Inst./ Inst. principal: Neurosciences, Mental Health and Addiction / Neurosciences, santé mentale et toxicomanies

Other Related Inst./ Autres inst. connexes: Gender and Health / Santé des femmes et des hommes; Health Services and Policy Research / Services et politiques de la santé; Population and Public Health / Santé publique et des populations

Competition Outcome/Résultats du concours: Team Grant: Mental Wellness in Public Safety Team Grants / Subv. d'équipe : Bien-être mental du personnel de la sécurité publique

November/Novembre 05, 2019

Number in competition/Nbre de demandes dans le concours: 16

Number approved/Nbre de demandes approuvées: 6

Decision on your application/ Décision sur votre demande: Approved / Approuvée

Average annual amount/ Montant annuel moyen: $286,667

Term/Durée: 3 **yrs/ans** 0 **months/mois**

Peer Review Committee Recommendation, for your information and use/ Recommandation du comité d'examen par les pairs, pour fins d'information et d'utilisation:

Committee/Comité: Team Grant: Mental Wellness in Public Safety Team Grants – Full Application / Sub. d'équipe: Sub. d'équipe sur le bien-être mental du personnel de la sécurité publique

Application rank within the competition/ Rang de la demande dans ce concours: 7

Percent Rank Within the Competition/ Rang en pourcentage au sein du concours: 43.75%

Rating/ Cote: 3.57

Recommended average annual amount/ Montant annuel moyen recommandé: $286,667

Additional Funding Opportunities/ Opportunités de financement additionnelles	Decision/ Décision	Total Funding Amount/Montant total du financement	Competition Code/Cote de concours	Application Number/ Numéro de la demande
Team Grant: Mental Wellness in Public Safety Team Grants - Correctional Services/Subv. d'équipe : Bien-être mental du personnel/sécurité pub.-serv. correctionnel	Not Approved/ Non approuvée	$0	201911MW4	440139

*** Applications receiving a score of less than 3.5 on any evaluation criteria will not be considered for Funding. / Les demandes qui ont reçu une note inférieure à 3.5 pour n'importe quel des critères d'évaluation ne sont pas admissibles.

This document is for information only. Official payment is stated on the CIHR Authorization for Funding.
Document à titre d'information seulement. Le paiement officiel est indiqué sur l'autorisation de financement des IRSC.

Canadian Institutes of Health Research
160 Elgin Street, 9th Floor
Address Locator 4809A
Ottawa, Ontario K1A 0W9

Instituts de recherche en santé du Canada
160, rue Elgin, 9e étage
Indice de l'adresse 4809A
Ottawa (Ontario) K1A 0W9

Institute of Aging
Institute of Cancer Research
Institute of Circulatory and Respiratory Health
Institute of Gender and Health
Institute of Genetics
Institute of Health Services and Policy Research
Institute of Human Development and Child and Youth Health
Institute of Indigenous Peoples' Health
Institute of Infection and Immunity
Institute of Musculoskeletal Health and Arthritis
Institute of Neurosciences, Mental Health and Addiction
Institute of Nutrition, Metabolism and Diabetes
Institute of Population and Public Health

Institut du vieillissement
Institut du cancer
Institut de la santé circulatoire et respiratoire
Institut de la santé des femmes et des hommes
Institut de génétique
Institut des services et des politiques de la santé
Institut du développement et de la santé des enfants et des adolescents
Institut de la santé des Autochtones
Institut des maladies infectieuses et immunitaires
Institut de l'appareil locomoteur et de l'arthrite
Institut des neurosciences, de la santé mentale et des toxicomanies
Institut de la nutrition, du métabolisme et du diabète
Institut de la santé publique et des populations

March 31, 2020

Dr. Nazanin Alavi
166 brock street
Kingston, Ontario
K7L 3E2

Dear Dr. Alavi:

On behalf of the Canadian Institutes of Health Research (CIHR) we are pleased to inform you that your recent application submitted to the Team Grant: Mental Wellness in Public Safety Team Grants entitled "Delivering online cognitive behavioural therapy (iCBT) to address mental health challenges in correctional officers and other public safety personnel." has been approved for funding.

Documentation pertaining to the review of your application can be found through ResearchNet. Please note that your Authorization for Funding will follow electronically.

As CIHR does not notify co-applicants of the decision, we ask that you inform those individuals involved, along with their research institutions (if different from your own), of the outcome of this application.

Should you require additional information, please contact the CIHR Contact Centre at support-soutien@cihr-irsc.gc.ca. Please do not contact the officers or members of the peer review committee.

Congratulations on your success in this competition.

Sincerely,

Nathalie Gendron

Nathalie Gendron, Ph.D.
Manager, Program Design and Delivery
Research Program Portfolio

Canada

CIHR IRSC
Discoveries for life / Découvertes pour la vie

Review Type/Type d'évaluation: Committee Member 1/Membre de comité 1
Name of Applicant/Nom du chercheur: Alavi, Nazanin
Application No./Numéro de demande: 433679
Agency/Agence: CIHR/IRSC
Competition/Concours: 2019-11-05 Team Grant: Mental Wellness in Public Safety Team Grants/Subvention d'équipe : Subventions d'équipe sur le bien-être mental du personnel de la sécurité publique
Committee/Comité: Team Grant: Mental Wellness in Public Safety Team Grants – Full Application/Sub. d'équipe: Sub. d'équipe sur le bien-être mental du personnel de la sécurité publique
Title/Titre: Delivering online cognitive behavioural therapy (iCBT) to address mental health challenges in correctional officers and other public safety personnel

---

Assessment/Évaluation:

This is a novel and much needed approach towards helping to ensure Correctional Officer wellness and mental health. As noted in the proposal, stigmatizing of officers who need, or should seek help is a major barrier towards them seeking assistance. In addition, the demands of shift work are an additional barrier towards seeking help. The proposal solves both of these issues via the use of on-line 24/7 availability for counseling / therapy.

Some of the difficulties the researchers may consider might involve the use of group therapy / discussions during phases of the research. The same reasons that officers avoid seeking help out of fear of being stigmatized could have a chilling effect on their willingness to participate in any type of group settings.

An additional consideration may involve the attrition of officers who are, or have committed, to being participants in the study. Typically, the attrition rate of correctional officers is rather high when compared to other public safety providers. Many leave for more “meaningful” work in the public safety sector and some quickly learn that they simply are “not cut out for” correctional work and return to safer, or more “stable” jobs with hours that promote, rather than detract from family life.

Review Type/Type d'évaluation: Committee Member 2/Membre de comité 2
Name of Applicant/Nom du chercheur: Alavi, Nazanin
Application No./Numéro de demande: 433679
Agency/Agence: CIHR/IRSC
Competition/Concours: 2019-11-05 Team Grant: Mental Wellness in Public Safety Team Grants/Subvention d'équipe : Subventions d’équipe sur le bien-être mental du personnel de la sécurité publique
Committee/Comité: Team Grant: Mental Wellness in Public Safety Team Grants – Full Application/Sub. d’équipe: Sub. d’équipe sur le bien-être mental du personnel de la sécurité publique
Title/Titre: Delivering online cognitive behavioural therapy (iCBT) to address mental health challenges in correctional officers and other public safety personnel

---

Assessment/Évaluation:

This proposal aims characterize unique mental health challenges in correctional workers and other public safety personnel; to develop therapy content to address unique mental health problems; to evaluate the efficacy of online CBT to improve access to care; and to evaluate the non-inferiority efficacy of online CBT compared to in-person CBT in improving mental health symptoms in this population. Overall, the application is well-written and the aims are well-developed and justified. Below, this reviewer provides comments according to the evaluation criteria for this review.

1. Impact of the Research

The proposed research has the potential to provide new knowledge regarding the unique mental health challenges of correctional workers and other public safety personnel, and to develop a tailored online CBT programme specific to this population. This approach is superior to using “off the shelf” online CBT programmes, as it targets the unique mental health needs of this population. The proposed aims are systematic and integrative, and follow established methods in the field.

The proposal integrates sex and gender, and will employ a gender-sensitive approach at all phases of the study. While there is relatively little consideration given to different population subgroups (e.g., ethnic, socioeconomic, sexuality), this may be related to the sociodemographic composition of the target population. Of note, the team does note that they will address the unique mental health needs of administrative and operational personnel separately.

The potential net result of the proposed research is a scalable online intervention that can address the unique mental health needs of correctional workers and other public safety personnel. Given that most online therapy studies are strictly research-based, the proposed research has the potential to be utilized more broadly in the psychological treatment armamentarium of mental health clinicians who serve this population.

2. Approach

The proposed design of the study is appropriate and will employ state-of-the-art methods to evaluate study aims. The literature review is complete and up-to-date, and demonstrates the team’s in-depth understanding of the broader scientific literature on the efficacy of online CBT for mental health problems. The roles and responsibilities of the Sex and Gender Champion identified adhere to the best practices outlined by the Institute of Gender and Health. The research team clearly articulated the limitations of their proposed study, as well as approaches to address them, most notably to address issues related to mental health stigma, which is among the most salient challenges to working with mental health issues in correctional workers.

Review Type/Type d'évaluation: Committee Member 2/Membre de comité 2
Name of Applicant/Nom du chercheur: Alavi, Nazanin
Application No./Numéro de demande: 433679
Agency/Agence: CIHR/IRSC
Competition/Concours: 2019-11-05 Team Grant: Mental Wellness in Public Safety Team Grants/Subvention d'équipe : Subventions d'équipe sur le bien-être mental du personnel de la sécurité publique
Committee/Comité: Team Grant: Mental Wellness in Public Safety Team Grants – Full Application/Sub. d'équipe: Sub. d'équipe sur le bien-être mental du personnel de la sécurité publique
Title/Titre: Delivering online cognitive behavioural therapy (iCBT) to address mental health challenges in correctional officers and other public safety personnel

Assessment/Évaluation:

One potential limitation is the extent to which the correctional workers who will be recruited into the study are representative of the broader population of correctional workers. Given issues of mental health stigma, do the researchers anticipate recruiting a healthier/sicker sample? How might this impact generalizability of results? What measures are in place to ensure broader generalizability of study results? Related to this point, the inclusion/exclusion criteria are not specified.

3. Originality of the Proposal

The originality of the proposal hinges on the unique approach to developing an online CBT programme that targets the unique mental health needs of correctional workers. The approach to developing the intervention is also original, as it takes a careful and systematic approach to identifying these mental health needs, and then using that information to develop and evaluate the efficacy of a target online CBT intervention.

4. Applicant(s)

The research team has an excellent track record of excellence in the proposed area. The PI is one of the pioneers of online CBT in Canada. The team exemplifies a team grant by including experts covering all of the relevant areas for this multidisciplinary project.

5. Open Science and Knowledge Translation Plan

The researchers outline a detailed knowledge translation and outcome impact plan that embraces open science principles and practices over the duration of the proposed study. The proposed dissemination of the online CBT programme has the potential to help improve mental health symptoms in the broader community of correctional workers across Canada.

What is somewhat unclear, however, is *how* the availability of the intervention will be made known to the broader community of correctional workers. And how can correctional workers trust the intervention such that their sensitive mental health data and perhaps lack of response to therapy and symptomatic status will not be shared with others, most notably their employer?

**Review Type/Type d'évaluation:**	Committee Member 2/Membre de comité 2
**Name of Applicant/Nom du chercheur:**	Alavi, Nazanin
**Application No./Numéro de demande:**	433679
**Agency/Agence:**	CIHR/IRSC
**Competition/Concours:**	2019-11-05 Team Grant: Mental Wellness in Public Safety Team Grants/Subvention d'équipe : Subventions d'équipe sur le bien-être mental du personnel de la sécurité publique
**Committee/Comité:**	Team Grant: Mental Wellness in Public Safety Team Grants – Full Application/Sub. d'équipe: Sub. d'équipe sur le bien-être mental du personnel de la sécurité publique
**Title/Titre:**	Delivering online cognitive behavioural therapy (iCBT) to address mental health challenges in correctional officers and other public safety personnel

Assessment/Évaluation:

Finally, one area that could be developed further is regarding the anonymity/confidentiality of enrollment in the online therapy program. Will this information ever travel back to an administrator? How might this affect the workers' employment, ability to carry weapons, etc? What if a highly symptomatic correctional worker is suicidal and requires more in-depth psychiatric care? What procedures are in place to help address these issues with what is essentially a "hands-off" intervention?

Review Type/Type d'évaluation: Committee Member 3/Membre de comité 3
Name of Applicant/Nom du chercheur: Alavi, Nazanin
Application No./Numéro de demande: 433679
Agency/Agence: CIHR/IRSC
Competition/Concours: 2019-11-05 Team Grant: Mental Wellness in Public Safety Team Grants/Subvention d'équipe : Subventions d’équipe sur le bien-être mental du personnel de la sécurité publique
Committee/Comité: Team Grant: Mental Wellness in Public Safety Team Grants – Full Application/Sub. d’équipe: Sub. d’équipe sur le bien-être mental du personnel de la sécurité publique
Title/Titre: Delivering online cognitive behavioural therapy (iCBT) to address mental health challenges in correctional officers and other public safety personnel

---

Assessment/Évaluation:

1. The sex/gender component of the application was done very well.
2. The plan for having CWs contribute to the design of the treatment programs is thorough. I particularly appreciated the material on peer-support.
3. The proposal is extremely repetitive. For example, the first 2 sentences of the research summary is repeated exactly in the subsequent background section on the same page! Eliminating this type of repetition could allow for the inclusion of other important details. Some of these details are noted below.
a. There are almost no details on the CBT to be provided. There are a lot of different cognitive and behavioral therapies, so some details are needed (e.g., will it include behavioral activation, schema work, exposure, cognitive work).
b. There is no specific plan to include trainees in a meaningful way.
c. What does multi-media involve (e.g., Will videos be used or will it mostly be like a series of powerpoint slides?).
4. The KT plan is brief and vague. Ideally, the applicants could demonstrate expertise (or past success) in the open science aspect of their proposal (e.g., preregistering a study).
5. The applicants have me convinced that there is stigma about mental health in CW. Unfortunately, this leads me to question the feasibility of conducting the initial interviews about this topic and the focus group interview.
6. Aim 4 is about comparing the non-i-CBT to iCBT. The sample sizes used might be sufficient to show people in both groups improve from time 1 to time 2. HOWEVER, they are not sufficient to provide an adequate test of equivalence or non-equivalence of the 2 therapies. With such a small sample size it is extremely likely that both treatments will be found to work about the same. This is my main concern regarding the research. Look at using a non-inferiority design.
7. I also question the appropriateness of the non-i-CBT as a comparison group. It seems like it is an in-person version of the new program developed for online use. While there may be good reason for using this type of CBT, it is certainly not the same as more standard approaches that participants might be able to access if they were not in the study (e.g., the type of CBT that a registered psychologist would use with the same client).
8. Why use 3 different methods of assessing depression (DASS-42, QIDS-SR 16, and PHQ-9)?
9. The proposal states that the applicants will “share all the therapy modules developed through this study.” More details of these materials will be shared is needed. Reviewers need to be convinced that a huge investment of money in this research will have a broad and long-term impact. If the modules are what is shared and this info can only really be used by clinicians not working online, then the study will have done little to address the barriers highlighted in the literature review.

Review Type/Type d'évaluation: SO Notes /Notes de l'agent scientifique
Name of Applicant/Nom du chercheur: Alavi, Nazanin
Application No./Numéro de demande: 433679
Agency/Agence: CIHR/IRSC
Competition/Concours: 2019-11-05 Team Grant: Mental Wellness in Public Safety Team Grants/Subvention d'équipe : Subventions d'équipe sur le bien-être mental du personnel de la sécurité publique
Committee/Comité: Team Grant: Mental Wellness in Public Safety Team Grants – Full Application/Sub. d'équipe: Sub. d'équipe sur le bien-être mental du personnel de la sécurité publique
Title/Titre: Delivering online cognitive behavioural therapy (iCBT) to address mental health challenges in correctional officers and other public safety personnel

---

Assessment/Évaluation:

The Committee recognized the value and importance of this study, particularly considering factors such as stigmatization associated with help seeking, and the challenges of accessing help under shift work conditions.
High staff turnover in this context was recognized as a challenge for research in this area.
The approach of assessing effectiveness of tailored online CBT was recognized as an approach with high potential for positive impact, with clear potential for superiority to an off-the-shelf version.
Integrated appropriate measures are in place and the approach would be scalable for implementation if the results are positive.
There were questions about representativeness of the sample in terms of extent of recruitment of healthier /sicker participants. Inclusion and exclusion criteria were not specified.
Although the applicants indicated an intention to focus on administrative and operational staff separately, the was no detailed discussion of analysis of responses in different subgroups (e.g. ethnic, socioeconomic, sex and gender), this may be related sample size.
There were some questions about how confidentiality will be maintained – of relevance to possible stigma effects. The committee also had questions about how adverse events may impact the study.
There was a clear knowledge translation plan.
Questions arose around client trust in relation to knowledge in the community about potential access to CBT and the issues of privacy in relation to participation and results will be maintained from the community and specifically, the employer.
The integration of sex and gender was well described and the presence of a gender champion on the team was recognized as a positive feature.
The research team was seen as excellent and highly productive.
